# Mitochondrial Quality Control: Decommissioning Power Plants in Neurodegenerative Diseases

**DOI:** 10.1155/2013/180759

**Published:** 2013-10-28

**Authors:** Rukmini Mukherjee, Oishee Chakrabarti

**Affiliations:** Structural Genomics Division, Saha Institute of Nuclear Physics, 1/AF Bidhannagar, Kolkata 700064, India

## Abstract

The cell has an intricate quality control system to protect its mitochondria from oxidative stress. This surveillance system is multi-tiered and comprises molecules that are present inside the mitochondria, in the cytosol, and in other organelles like the nucleus and endoplasmic reticulum. These molecules cross talk with each other and protect the mitochondria from oxidative stress. Oxidative stress is a fundamental part of early disease pathogenesis of neurodegenerative diseases. These disorders also damage the cellular quality control machinery that protects the cell against oxidative stress. This exacerbates the oxidative damage and causes extensive neuronal cell death that is characteristic of neurodegeneration.

## 1. Introduction

Mitochondria are key molecular players in all cells performing many vital functions. They are the powerhouse of the cell, providing the cell with adenosine triphosphate (ATP) generated by oxidative phosphorylation (OXPHOS). Mitochondria have an important role in buffering calcium flux from the endoplasmic reticulum and the plasma membrane thus helping to maintain the spatiotemporal distribution of calcium in the cell. Mitochondria have enzymes essential for steroid synthesis and are the chief source of endogenous reactive oxygen species (ROS) which are produced by several mitochondrial enzymes including components of the OXPHOS system [1].

Proper mitochondrial functioning is very important to neurons. Neurons have high energy requirements. They are terminally differentiated cells which consume a lot of ATP to maintain ion gradients across membranes for proper neurotransmission. Efficient mitochondrial transport and positioning are also critical because different regions of the neuron have different energy requirements. Active growth cones and presynaptic terminals need more ATP than other parts of the cell [2]. Calcium buffering by mitochondria is important to neurons. Presynaptic mitochondria are responsible for clearing calcium for proper neurotransmitter release and can affect the rate of recovery from synaptic depression after moderate synaptic activity [3]. Also, neurons have lipid membranes with high proportions of polyunsaturated fatty acids which are susceptible to oxidative damage by reactive oxygen species. Therefore, neuronal functioning relies heavily on the presence of healthy mitochondria, and consequently mitochondrial dysfunction is a fundamental part of neurodegeneration. Impairment of the vital functions of the mitochondria broadly referred to as “mitochondrial dysfunction” causes the cell to take protection against stress by activating a multitiered defence system which involves not only the mitochondria but also other cellular machinery like the cytoplasmic ubiquitin proteasomal system (UPS), the autophagy process, part of the endoplasmic reticulum quality control machinery, and finally activation of programmed cell death as the last level of defence. This review summarises the response of the cellular quality control machinery to mitochondrial damage associated with neurodegenerative disease and the alterations caused to these cellular surveillance systems in common neurodegenerative disorders.

## 2. Oxidative Stress and Neurodegeneration

Mitochondria are the main producers of endogenous reactive oxygen species. ROS are an inevitable by-product of oxidative phosphorylation. Mitochondrial enzymes that generate ROS include the members of the electron-transport chain (ETC): complexes I, II, and III; tricarboxylic acid (TCA) cycle enzymes aconitase and *α*-ketoglutarate dehydrogenase; pyruvate dehydrogenase; glycerol-3-phosphate dehydrogenase; dihydroorotate dehydrogenase; the monoamine oxidases; and cytochrome *b*_5_ reductase [1]. ROS levels in the mitochondrial matrix are determined by the proton gradient across the inner membrane, the efficiency of ATP production by the respiratory chain, and the ratio of the reduced to oxidised form of nicotinamide adenine dinucleotide (NADH/NAD^+^ ratio) [4]. ROS can cause oxidative damage to mitochondrial proteins, mutations in mitochondrial DNA (mtDNA), oxidation of lipids in the mitochondrial membranes, opening of the mitochondrial permeability transition pore, and release of proapoptotic molecules like cytochrome c from the mitochondria. Excess ROS production and oxidative damage can operate in a vicious cycle where one can trigger the other. Mitochondria have antioxidants like glutathione and *α*-tocopherol and enzymes like manganese superoxide dismutase (MnSOD), catalase, and glutathione peroxidase to detoxify ROS. However, perturbation of the delicate balance between the antioxidant defence capacity and the ROS levels leads to oxidative stress and mitochondrial damage.

**Figure 1:**
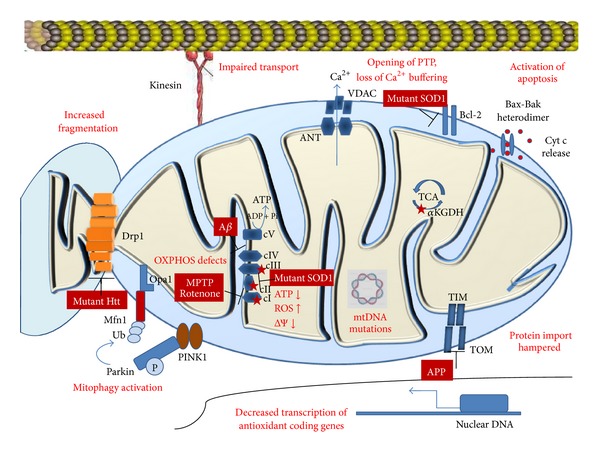
Mitochondrial changes in neurodegeneration. Most neurodegenerative diseases result in defects in oxidative phosphorylation, respiratory dysfunction, increased ROS production, lowered mitochondrial membrane potential, decrease in synthesis of antioxidants, mtDNA mutations, impaired protein import, increased fragmentation of mitochondria, and activation of mitophagy and apoptosis. Sources of reactive oxygen species are marked by red stars. Abbreviations: Htt, Huntingtin; Ub, ubiquitin; ΔΨ, membrane potential; MPTP, 1-methyl-4-phenyl-1,2,3,6-tetrahydropyridine; ROS, reactive oxygen species; mtDNA, mitochondrial DNA; A*β*, amyloid beta; APP, amyloid precursor protein; TIM, translocase of inner membrane; TOM, translocase of outer membrane; SOD1, superoxide dismutase 1; VDAC, voltage dependent anion channel; ANT, adenine nucleotide translocator; Pink1, PTEN-induced putative kinase 1; *α*KGDH, alpha ketoglutarate dehydrogenase; cyt c, cytochrome c.

Oxidative damage is a fundamental part of the pathophysiology of neurodegenerative diseases (Figure 1). Oxidative damage has been noted in postmortem brain slices from patients with moderate and late stage Alzheimer's disease (AD). Levels of antioxidants like glutathione and activity of enzymes like catalase and superoxide dismutase were low in the frontal cortex of AD patients [5]. In Huntington's disease (HD) model, striatum and cortex of mice expressing full length endogenous mutant Huntingtin (Htt^140q/140q^) have higher activity of NADPH oxidase which generates ROS than in similar aged controls [6]. In familial amyotrophic lateral sclerosis (ALS), mutations in the gene encoding copper/zinc superoxide dismutase lead to increased levels of ROS and oxidative damage. The role of oxidative stress is also well characterised in Parkinson's disease (PD). Auto-oxidation of dopamine to quinones generates hydrogen peroxide which increases oxidative stress in the PD brain. Also, increased levels of Fe^3+^ and ferritin have been noted in the substantia nigra of Parkinson's disease patients which contributes to oxidative stress and neuronal death. Mutations in Parkinson's disease associated genes like those encoding Parkin, PINK1 (PTEN-induced putative kinase 1), HTRA2 (high temperature requirement protein A2), and DJ-1 affect mitochondrial morphology and function and cause oxidative stress [7]. Oxidative damage has also been noted in transmissible spongiform encephalopathies (TSEs). Conversion of cellular prion protein (Prp^C^) to proteinase K-resistant prion protein (Prp^Sc^) correlates with respiratory dysfunction and changes in mitochondrial ultrastructure [8]. Some of the neurodegenerative diseases are characterised by formation of protein aggregates. These aggregates are not metabolically inert but actively alter cellular metabolism increasing oxidative stress. Oxidatively modified proteins escape proteasomal degradation seeding the formation of more aggregates [9]. 

## 3. Mitochondrial Quality Control

The mitochondria evolved from the *α*-proteobacteria which was engulfed by a preeukaryote 1.5 billion years ago. As a result, it is a semiautonomous organelle which is bounded by a double membrane system, has its own genome which codes for a small subset of its proteins, and has a few components of the protein synthesis machinery. However for its complete functioning, it is dependent on proteins encoded by the nuclear genome, cellular enzymes, protein synthesis machinery, and signalling molecules.

The quality control system residing in the mitochondria is also semiautonomous like other aspects of its functioning. It is an essential but inadequate system to protect the organelle from stress. The intraorganellar surveillance system comprises molecules distributed over all the compartments of the organelle: the innermost matrix, the inner membrane (thrown into folds called cristae), the intermembrane space, and the outer membrane. These molecules are a group of chaperones and proteases which help in proper folding of mitochondrial proteins and removal of oxidatively damaged proteins from the mitochondria. Antioxidant enzymes residing in the matrix can also be considered to be a part of the quality control system. They are important scavengers of reactive oxygen species produced by the respiratory chain. The next level of quality control comes from the dynamic nature of the mitochondria. Constant fission-fusion processes help in repair of slightly damaged or depolarised mitochondria or help to segregate out mitochondria which are beyond repair. The rest of the stress defence system is extramitochondrial, consisting of the cytosolic ubiquitin proteasomal system and the cellular autophagy machinery. Recent reports show that interorganellar communication and contacts with the endoplasmic reticulum can also protect mitochondria from oxidative stress and can induce mitophagy [10]. Hence, protection of the cell and its mitochondria during oxidative stress is an integrated effort of the mitochondrial and several cellular quality control mechanisms. These systems communicate and cross talk with one another to guard the cell against oxidative stress. They are also linked to the molecular players of the apoptosis cascade which gets triggered when the quality control system fails to protect the cell from redox stress. These defences against oxidative stress get altered in neurodegeneration leading to extensive oxidative damage and neuronal cell death.

### 3.1. The Intramitochondrial Defence System

**Figure 2:**
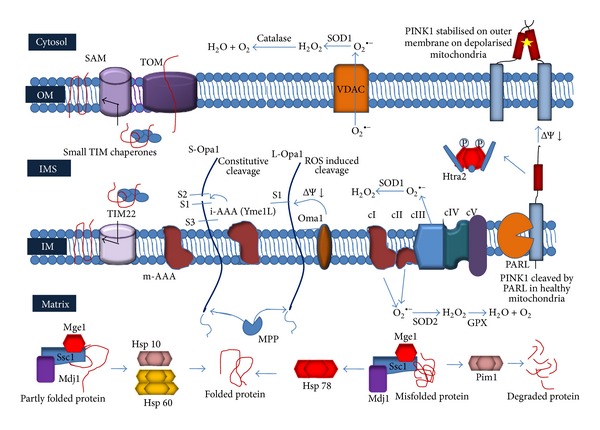
The intramitochondrial quality control system. The intraorganellar quality control system comprises antioxidant enzymes, molecular chaperones, and proteases found in all compartments of the mitochondria. OM, mitochondrial outer membrane; IM, mitochondrial inner membrane; IMS, intermembrane space.

The intramitochondrial defence against oxidative damage includes antioxidants which scavenge the ROS right at its source of production, chaperones which refold misfolded or oxidatively modified proteins, and proteases like the Lon proteases and caseinolytic peptidase X (ClpXP) which can degrade damaged proteins which are beyond repair (Figure 2).

#### 3.1.1. Antioxidant Defence Capacity of the Mitochondria

ROS are needed by cells at physiological levels. They have important regulatory roles in several signalling cascades. These include the mitogen-activated protein kinase (MAPK) pathway in cytokine signalling [11], nuclear factor kappa-light-chain enhancer of activated B cells (NF*κ*B) signalling in response to increased hydrogen peroxide (H_2_O_2_) levels [12], and c-Jun N-terminal kinase (JNK) signalling [13]. Signalling mediated by ROS often activates transcription of genes coding for antioxidants. On exposure to ROS, nuclear factor (erythroid-derived 2) like 2 (Nrf2) translocates into the nucleus, binds the antioxidant response element (ARE) in the promoter of antioxidant coding genes, and upregulates their transcription. Kelch like-ECH-associated protein 1 (Keap1) can sequester Nrf2 in the cytosol and help in maintaining redox balance [14]. These mechanisms to maintain redox homeostasis get hampered in neurodegenerative diseases. Nuclear level of Nrf2 is lowered in hippocampal neurons of Alzheimer's disease patients [15]. In Parkinson's disease (PD), though Nrf2 translocates to the nucleus, levels of ARE responsive genes like quinone oxidoreductases (NQOs) and heme oxygenases (HOs) are lower in neurons of the substantia nigra than in glial cells [16]. 

Antioxidants like glutathione (GSH) and thioredoxin protect the mitochondria against oxidative stress. The thioredoxin system is important for maintaining redox homeostasis. It consists of two oxidoreductases—thioredoxin reductase (TrxR) and thioredoxin (Trx)—which work together to reduce disulfide bonds in substrate proteins. Apart from its direct antioxidant function, thioredoxin's interaction with apoptosis signal-regulating kinase 1 (ASK1) can modulate gene expression of p38 MAPK and JNK [17]. GSH nonenzymatically scavenges free radicals like superoxide, nitric oxide, hydroxyl radical, and peroxynitrite. It works as an electron donor in the reaction catalysed by glutathione peroxidase which reduces H_2_O_2_ to water. Levels of glutathione decrease in dopaminergic neurons of the substantia nigra pars compacta (SNpc). This decrease occurs early in disease pathogenesis and is even seen in presymptomatic Parkinson's disease or incidental Lewy body disease [18]. Dopamine may cause upregulation of GSH synthesis. Blocking an enzyme in the dopamine synthesis pathway prevented increase in glutathione levels in SHSY5Y cells. Dopamine may upregulate transcription of genes involved in glutathione synthesis and release [19]. Antioxidant activity is also provided by detoxifying enzymes like superoxide dismutases (the cytosolic superoxide dismutase (SOD1) and the mitochondrial isoform (SOD2)), catalase (Cat), glutathione peroxidase (GPX), phospholipid hydroperoxide glutathione peroxidase (PGPX), glutathione reductase (GR), peroxiredoxins (PRX3/5), glutaredoxin (GRX2), thioredoxin (TRX2), and thioredoxin reductase (TRXR2). Activity of these enzymes gets disrupted in oxidative stress associated with neurodegenerative disease. SOD1 mutations are well known in amyotrophic lateral sclerosis—a disease characterised by loss of motor neurons of the central nervous system. Recent studies on ALS show that overexpression of the mitochondrial superoxide dismutase (SOD2) can decrease SOD1 associated cytotoxicity and cell death in human neuroblastoma cell line LAN5 expressing mutant SOD1 [20]. Amyloid precursor protein (APP) mutant/MnSOD heterozygous knockout (APP^19959^/MnSOD^+/-^) mice show increased levels of Amyloid beta in Alzheimer's disease model [21]. Lowered activity of ROS detoxifying enzymes like superoxide dismutase and catalase has been shown in knock-out mice lacking cellular prion protein [22]. In fact, the physiological function of Prion protein has been predicted to be that of an antioxidant [22, 23].

#### 3.1.2. Mitochondrial Chaperones

Mitochondrial chaperones include heat shock proteins (HSPs) like members of Hsp60, Hsp70, and Hsp100 family of chaperones. Their classification is based on molecular weight, but they have different structural features and also have distinct roles in the mitochondria. Hsp70 family members that reside in the mitochondrial matrix like Stress-Seventy subfamily C1 (Ssc1) in *Saccharomyces cerevisiae* help in translocation and folding of precursor proteins imported into the mitochondria. Ssc1 works in an ATP dependent manner with cochaperones Mitochondrial DnaJ1 (Mdj1) and Mitochondrial GrpE1 (Mge1) which assist in substrate interaction and nucleotide exchange, respectively, [24]. Small TIM chaperones are another set of chaperones which are present in the intermembrane space and help in translocation and beta barrel formation of mitochondrial membrane proteins by interacting with the translocase of the outer membrane (TOM), sorting and assembly machinery (SAM) supercomplex [25]. Heat shock protein 78 (Hsp78) in yeast is an Hsp100/Clp family chaperone which can protect the mitochondria from thermal stress by causing disaggregation and refolding of damaged proteins. It can also work with proteases like Pim1 to degrade misfolded proteins. Studies by Bender et al. have identified eight mitochondrial proteins which are aggregation prone at high temperatures. They have used temperature sensitive Hsp mutants of yeast to study the protective chaperone activity of mitochondrial Hsp70 (mtHsp70 or Ssc1) in preventing aggregation of two aggregation-prone proteins—aconitase (Aco1) and acetolactate synthase (Ilv2) [26]. Molecular chaperones of the mitochondria have recently been linked to neurodegenerative disorders. A proteomic approach showed that mtHsp70 or Mortalin interacts with DJ1—a protein involved in oxidative stress related to Parkinson's disease. Mutational analysis of German Parkinson's disease (PD) patients identified polymorphisms in the coding region of the *mortalin* gene. These variants of the Mortalin protein can cause mitochondrial dysfunction in PD [27]. Cytoplasmic chaperones also aid in transport of mitochondrial precursor proteins to the mitochondria. Complex I subunits coded by the nucleus are escorted to the mitochondria by the chaperone heat shock protein 90 (Hsp90) and Sicily a homologue of C8ORF38—a chaperone whose loss causes Leigh syndrome. Loss of Sicily leads to faulty import of complex I subunits and neurodegeneration [28].

#### 3.1.3. Mitochondrial Proteases

Mitochondrial proteases have two important functions. Some proteases like the processing peptidases are important in mitochondrial biogenesis, while the other group of proteases are involved in mitochondrial quality control.

Processing peptidases cleave sorting signals of nucleus-encoded precursor proteins upon import into the mitochondria. These include mitochondrial processing peptidases (MPPs), mitochondrial intermediate peptidase (MIP), and inner membrane peptidases (IMPs). Some processing peptidases like presenilin-associated rhomboid-like protease (PARL) have regulatory roles. PARL is one of the proteases which cleave Optic atrophy 1 (Opa1)—the mitochondrial inner membrane protein responsible for inner membrane fusion. Cleavage of Opa1 from its long to short isoform is needed for proper fusion activity of the mitochondria and can protect cells from apoptosis. However, PARL is dispensable for Opa1 processing as cells lacking PARL show normal Opa1 cleavage [29]. 

When antioxidants and molecular chaperones fail to protect mitochondrial proteins from oxidative damage, intraorganellar proteases distributed in all the compartments of the mitochondria degrade the damaged proteins. These may be ATP dependent or ATP independent in their functioning. ATP dependent proteases include the Lon proteases (Pim1 in yeast) and caseinolytic peptidases (ClpP) in the matrix, and the AAA+ family of proteases which are mostly present on the inner membrane. Depending on whether they are catalytically active on the matrix side or intermembrane side of the mitochondrial inner membrane, they are subdivided into m-AAA proteases and i-AAA proteases. Proteomic analysis of isolated mitochondria of *Saccharomyces cerevisae* subjected to oxidative stress showed that the major subset of proteins that are susceptible to ROS mediated degradation are enzymes involved in the detoxification of oxygen radicals and proteins with iron-sulfur clusters. This study also identified Pim1 to be the major mitochondrial protease that degrades proteins in response to enhanced oxidative stress [30]. AAA+ proteases like the YME1-like 1 ATPase (YME1L1) regulate Opa1 cleavage. This cleavage of Opa1 is dependent on the membrane potential of the mitochondria. Some of these Opa1 processing proteases have overlapping functions and can substitute for one other. For example, the m-AAA proteases AFG3 (ATPase family gene 3)-like 1 (AFG3L1) and AFG3L2 can carry out the function of Paraplegin. However, neither Paraplegin nor AFG3L2 is completely dispensable as mutations in the gene encoding Paraplegin cause a recessive form of hereditary spastic paraplegia, whereas heterozygous mutations in the gene encoding AFG3L2 cause a dominant form of spinocerebellar ataxia. Oligopeptidases like the HtrA serine peptidase 2 (HtrA2/Omi) are present in the intermembrane space of mitochondria and are released into the cytosol in response to apoptotic stimuli. Once in the cytosol, it antagonises inhibitors of apoptosis (IAPs), as a result, caspases are activated which result in apoptotic cell death [31]. A missense mutation in the protease domain of HtrA2 (mnd2 mutation) can cause neuromuscular disorder with striatal neuron degeneration [32]. HtrA2 has been found to interact and process amyloid precursor protein (APP) in mouse brains and in cultured cells without any apoptotic stimuli [33]. Therefore, mitochondrial proteases have an important housekeeping role in precursor protein processing and a quality control function during oxidative stress. Exact molecular mechanisms, specific substrates, and effects of most mitochondrial proteases remain unclear, but some of these proteases have been linked to oxidative stress and neurodegenerative disorders.

### 3.2. Quality Control by Regulating Mitochondrial Dynamics

Mitochondria are dynamic organelles. They constantly divide and fuse with one another, move within the cell on microtubule or actin tracks, and show changes in shape and ultrastructure. 

Mitochondrial fission-fusion events make use of proteins on the inner and outer mitochondrial membrane. Fusion is governed by the Mitofusin (Mfn) proteins on the outer membrane (Mfn1 and Mfn2) which tether adjacent mitochondria to each other and Optic atrophy 1 (Opa1) on the inner membrane which interacts with Mfn1 on the outer membrane and helps in inner membrane fusion of mitochondria. Fission processes are controlled by Dynamin related protein 1 (Drp1), Fission 1 (Fis1), and other proteins. Drp1 is a large dynamin like GTPase which oligomerises on the mitochondrial outer membrane to form a ring like structure which constricts the outer membrane to cause fission. Fission 1 (Fis1), Mitochondrial fission factor (Mff), and Mitochondrial dynamic protein homologs (MiD49 and MiD51) have been proposed to act as receptors that recruit Drp1 and may help in Drp1 assembly but the exact mechanism of Drp1 mediated mitochondrial fission remains unknown [34]. 

Mitochondrial dynamics is a finely controlled process. The fission-fusion balance can get altered depending on the metabolic status of the cell like presence of oxidative stress or conditions that induce autophagy. These stimuli can have different effects on mitochondrial dynamics depending on factors like the cell type or intensity of the stimulus [35]. Regulation of mitochondrial dynamics occurs mostly at the posttranslational level, as these responses in presence of stimuli like oxidative stress have to be fast events which would not need change in gene expression. Drp1 undergoes several posttranslational modifications like phosphorylation, SUMOylation, and ubiquitination. Phosphorylation of Drp1 at Ser^637^ occurs during starvation induced autophagy and in MEFs treated with Rapamycin [36]. SUMOylation of Drp1 has been noted in a few studies, and the sites at which SUMOylation occur have been identified, but the biological role of this modification is still unclear [37]. Ubiquitination of mitofusins and Drp1 have been shown to regulate mitochondrial dynamics. The mitochondrial E3 ligase Membrane-Associated Ring Finger (C3HC4) (MARCH5) ubiquitinates Drp1 and mitochondria become elongated and tubular on its overexpression [38]. However, in another study, it was seen that knockdown of MARCH5 causes formation of elongated mitochondria which produce higher levels of ROS and have lowered membrane potential when compared to control cells [39]. Also, altered mitochondrial dynamics can lead to loss of mitochondrial DNA. 

This dynamic nature of the mitochondria is essential for its function in quality control. Fission helps to separate out damaged mitochondria from the healthy interconnected mitochondrial network so that it can be removed by autophagy. Fusion on the other hand can help in exchange of mitochondrial proteins, mitochondrial DNA, and restore membrane potential of depolarised mitochondria. Inhibition of inner membrane fusion of completely depolarised mitochondria which are beyond repair is ensured by Opa1 inactivation [40]. Opa1 is inactivated by cleavage by a protease called overlapping with the m-AAA Protease 1 (Oma1) in a membrane potential dependent manner [41]. In such damaged mitochondria, the outer membrane fusion machinery also gets inactivated by ubiquitination of mitofusins Mfn1 and Mfn2. This effect is induced by Parkin and causes proteasomal degradation of Mfn1 and Mfn2 [42]. When protective mechanisms fail, a shift of the mitochondrial dynamics towards excess fission can induce apoptosis. Inhibition of Drp1 has been shown to prevent Staurosporine induced apoptosis and mitochondrial fragmentation in COS7 cells [43]. Changes in mitochondrial shape, size, and ultrastructure like cristae length and arrangement (called cristae remodelling) often occur in presence of apoptotic stimuli. Depolarising the mitochondria with uncouplers like carbonyl cyanide m-chlorophenyl hydrazone (CCCP) cause swelling, disruption, or loss of cristae [44]. Mitochondria can even fuse with the endoplasmic reticulum at the ER-mitochondria interface called mitochondria associated membranes (MAMs). This has an important role in maintaining calcium homeostasis.

Fission-fusion defects are prevalent in common neurodegenerative disorders. Mutations in Mfn2 cause Charcot Marie Tooth Decay type 2, and Opa1 mutations occur in dominant optic atrophy. Posttranslational modifications like S nitrosylation of Drp1 leading to increased fission occur in Alzheimer's disease model [45]. In studies on Huntington's disease pathogenesis, overexpression of mutant Huntingtin with 74 CAG repeats in HeLa cells leads to mitochondrial fragmentation and reduced ATP levels. This can be reversed by exogenous expression of dominant negative Mfn2 or Drp1 [46]. Such defects in the fission-fusion machinery hamper one level of mitochondrial quality control increasing chances of mitochondrial damage and dysfunction.

### 3.3. Mitophagy

When individual mitochondria fail to combat oxidative stress by using their own quality control machinery or by fusing and exchanging contents with healthy mitochondria, they have to be removed by the process of mitochondria selective autophagy called mitophagy. Autophagy can be of 3 types—chaperone mediated autophagy, microautophagy, and macroautophagy [47]. Mitophagy is generally considered to be a type of macroautophagy though electron microscopy studies in *Saccharomyces cerevisae* show that certain conditions like a nonfermentable carbon source can cause yeast cells to activate mitochondria selective microautophagy [48].

Macroautophagy consists of the following steps: the formation of an isolation membrane, elongation, closure of the isolation membrane around the cargo to form the autophagosome, and finally fusion of the autophagosome with the lysosome where the cargo gets degraded. Each step of this process needs a group of proteins which are coded by autophagy related genes (ATGs) [47].

The most pertinent question about mitophagy is its selectivity. How the autophagy machinery is selectively recruited to damaged mitochondria without activating bulk autophagy in the cell is not understood. Studies on mitophagy are mostly on mammalian cell lines and in *Saccharomyces cerevisae*, but what is surprising is that the components of mitochondria specific autophagy are not very conserved. Atg32 is a mitophagy specific receptor which is necessary for induction of mitophagy in yeast [49]. Atg32 on the mitochondrial outer membrane gets phosphorylated on Ser^114^ and interacts with Atg11 which helps to physically link the mitochondria to the isolation membrane [50]. No mammalian homologues of the yeast Atg32 have been discovered. In mammals, Nix is a mitochondrial protein which can directly bind the autophagosome marker microtubule-associated protein 1A/1B-light chain 3 (LC3) and help in mitochondria specific autophagy. Nix mediated autophagy occurs in differentiation of mature red blood cells. Mitochondrial depolarisation with CCCP treatment can activate mitophagy and can cause LC3 recruitment to the mitochondria [51]. Mitochondrial ROS which is released in short bursts can act as signalling molecules to induce mitophagy. When ROS levels are increased using a mitochondrial-targeted photosensitizer construct called mitochondrial KillerRed (mtKR), there is membrane depolarisation followed by activation of mitophagy mediated by the PINK1-Parkin pathway. Overexpression of the antioxidant enzyme Manganese superoxide dismutase scavenged ROS and hence prevented induction of mitophagy [52].

The PINK1-Parkin pathway of mitophagy is well studied, and mutations in PTEN induced putative protein kinase 1 (PINK1) and Parkin are common in early onset forms of juvenile Parkinson's disease. PINK1 is a kinase which gets stabilised on the mitochondrial surface when membrane potential is lowered and causes recruitment of the E3 ligase, Parkin, to depolarised mitochondria. Recently, Mfn2 was shown to be the Parkin receptor during mitophagy. PINK1 phosphorylates Mfn2. Phosphorylated Mfn2 acts as a receptor for binding of Parkin. Parkin ubiquitinates Mfn2 and marks the mitochondria for mitophagy. This is followed by accumulation of p62 puncta on the mitochondria followed by execution of mitophagy [53]. The PINK1-Parkin pathway also helps to quarantine depolarised mitochondria from being transported to energy requiring regions of the cell. PINK1 phosphorylates Miro which acts as a signal for Parkin to ubiquitinate and degrade Miro. As a result, kinesin is removed from the mitochondrial surface. So, depolarised mitochondria fall off the microtubule track and are not transported to energy requiring regions of the cell [54].

Mitochondrial dynamics often works in conjunction with mitophagy, where fission helps to segregate out damaged mitochondria so that they can be removed by mitophagy. Lowered membrane potential inactivates fusion proteins like Opa1 and mitofusins shifting the balance towards fission. Twig et al. showed that double labelling mitochondria with the potentiometric dye tetramethylrhodamine ethyl ester (TMRE) and mito-photoactivatable Green fluorescent protein (mito-PAGFP) can be used to determine change in membrane potential after fission. In most cases, fission forms two mitochondria of unequal membrane potential—one of which is depolarised, the other being hyperpolarised compared to prefission potentials. Depolarised mitochondria do not fuse and are removed by autophagy [55].

Mitophagy defects are seen in Parkinson's disease model where there are mutations in PINK1 and Parkin. Autophagy of mitochondria is increased in pyramidal neurons of Alzheimer's disease patients when compared to controls. Ultrastructure analysis showed cytochrome oxidase I to be in the cytosol and mitochondrial DNA to be present in lipofuscin containing vacuoles which are believed to be sites of autophagic turnover of mitochondria [56].

### 3.4. The Ubiquitin Proteasomal System in Mitochondrial Quality Control

The cytoplasmic ubiquitin proteasomal system has an important role in quality control of mitochondrial proteins. A lot of mitochondrial proteins have been found to be ubiquitinated and degraded by the proteasome. Mitochondrial proteins degraded by the proteasomal machinery include precursor proteins which are encoded by the nuclear genome and are misfolded or mistargeted during import into the mitochondria. This prevents buildup of defective proteins in the cytosol hence serving as a quality control mechanism [57]. Ubiquitination can alter mitochondrial dynamics. Sumoylation and ubiquitination can have opposite effects. Sumoylation of Drp1 induces mitochondrial fission, while ubiquitination of Drp1 leads to proteasomal degradation of Drp1 hence shifting the balance towards fusion [58, 59]. Ubiquitination of mitochondrial proteins can occur in stress, like loss of membrane potential, which causes Parkin dependent ubiquitination of Mfns [42].

Ubiquitination of mitochondrial proteins is carried out by cytosolic E3 ligases like Parkin which is recruited to the mitochondria upon depolarisation, by the F-box containing E3 ligase mitochondrial distribution and morphology 30 (Mdm30) which degrades Fzo1 (mitofusin homolog in* Saccharomyces cerevisae*), or by mitochondrial E3 ligases like MARCH5 and MULAN. Recent reports show that MITOL ubiquitinates mitochondrial Mfn2 (but not ER Mfn2) and causes its oligomerisation but not proteasomal degradation. Ubiquitination of Mfn2 by MITOL may hamper ER mitochondria interactions at the mitochondria associated membranes [60]. MITOL has also been shown to ubiquitinate Drp1, hFis1, and mutant SOD1 [61]. Blocking the proteasome causes accumulation of intermembrane space proteins like EndonucleaseG (EndoG). Ubiquitination of these proteins occurs prior to import into the organelle as deletion of the mitochondrial targeting sequence does not affect ubiquitination. On proteasome inhibition, the intermembrane space protease Omi/Htra1 cleaves endoG acting as a backup mechanism of protein quality control in the mitochondria when the proteasomal system is malfunctioning [62]. Deubiquitinating enzymes like Ubiquitin-specific processing protease 16 (Ubp16) have also been discovered to be present on the mitochondrial outer membrane [63].

Though most of these are mitochondrial outer membrane proteins, many matrix resident proteins are also ubiquitinated by the cytosolic UPS. A recent proteomic study has identified a wide array of interactors for the cytosolic E3 ligase PARKIN; along with cytosolic and nuclear molecular partners, surprisingly, there are also proteins of the mitochondrial matrix [64]. How the proteasomal machinery gets access to the proteins residing inside the mitochondria remains uncertain. Whether the proteasomal components are recruited to the mitochondria or other ancillary proteins extract the membrane proteins from the mitochondrial membrane and transport them to the proteasome is unknown [57]. 

Recent reports indicate the presence of a quality control mechanism similar to the endoplasmic reticulum associated degradation (ERAD) in mitochondria—termed mitochondria associated degradation (MAD). This mechanism involves retro translocation of proteins from the mitochondria to the cytosol where it can be degraded by the proteasome. This hypothesized mechanism is supported by several studies [65]. Valosin-containing protein (p97/VCP) is an AAA-ATPase and presents ERAD substrates to the proteasome. It has been shown that p97 is recruited to the mitochondria for Parkin mediated degradation of mitofusins, indirectly supporting the existence of the MAD mechanism. Studies in *Saccharomyces cerevisae* show that an interactor of VCP called VCP/Cdc48-associated mitochondrial stress-responsive 1 (Vms1) is recruited to the organelle during stress like loss of mitochondrial DNA or with CCCP treatment. Vms1 causes translocation of a small fraction of cellular VCP to the mitochondria. Vms1 is essential for proteasomal degradation of Fzo1 [66]. Further, gp78, an E3 ligase which spans the ER membrane and is a component of the ERAD, is found to interact with and degrade Mfn1 on the mitochondria. This can induce mitophagy in cells where the mitochondria is depolarised with CCCP. This induction of mitophagy is independent of Parkin, as it occurs in HeLa cells which are Parkin null or have very low expression levels of Parkin [10]. 

Neurodegenerative diseases often have accumulation of protein aggregates as part of their pathogenesis. Accumulation of ubiquitin conjugates and/or inclusion bodies is seen as the neurofibrillary tangles of Alzheimer's disease, Lewy bodies in Parkinson's disease, Bunina bodies in Amyotrophic Lateral Sclerosis, clear inclusions in CAG repeat expansion disorders like Huntington's disease, and extracellular amyloid aggregates in prion diseases. In some cases, the ubiquitin proteasomal system directly gets affected in these diseases. For example, mutations in the E3 ligase Parkin occur in Autosomal recessive Parkinson's disease. An E3 ligase called Mahogunin interacts with cytosolic Prion protein and is implicated to be involved with the pathophysiology of Prion diseases [67]. In other cases, the effect on the UPS is a secondary one; that is, the protein aggregates formed overload or inhibit the proteasome. Oxidative stress in neurodegeneration also affects the proteasomal degradation process. Mitochondrial ROS cause oxidative damage to the 19S regulatory particle of the 26S proteasome affecting proteasomal activity [68]. Also, ubiquitination and proteasomal degradation require ATP which decreases in mitochondrial dysfunction. Therefore, oxidative stress and proteasomal overload/dysfunction are common features of neurodegeneration. These two features act synergistically to enhance the degree of neuronal dysfunction and cell death.

## 4. Conclusion

**Figure 3:**
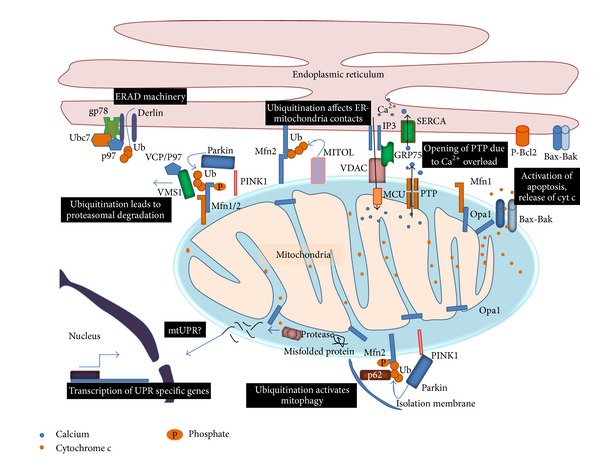
Integrated response of the cell to oxidative stress. In oxidative stress, the mitochondrial quality control works in sync with other cellular defences like the ubiquitin proteasomal system and the autophagy machinery. This involves interorganellar cross talk of the mitochondria with the endoplasmic reticulum and the nucleus. Failure of quality control systems leads to activation of apoptosis.

Oxidative stress is a characteristic feature of most neurodegenerative diseases. The chief cause of increased oxidative stress is often mitochondrial dysfunction, which leads to increased levels of reactive oxygen species. The cell has an intricate surveillance system to protect its mitochondria from oxidative stress. Part of this quality control machinery lies inside the mitochondria—a group of antioxidant enzymes which tries to prevent buildup of ROS beyond permissible levels, chaperones to refold misfolded proteins, and proteases to degrade damaged proteins. However, quality control against oxidative damage is not restricted to the mitochondrion itself but is an integrated effort which involves a bidirectional cross talk between the entire mitochondria network and communication with other cellular quality control machineries like autophagy and the ubiquitin proteasomal system. Recent research also shows the importance of interorganellar cross talk where the endoplasmic reticulum and the nuclear gene expression control have a prominent role in regulating mitochondrial quality control (Figure 3) [69].

Mitochondrial quality control is a relatively new field of research which has a lot of unanswered questions. A simple analysis of the events that follow when cells are treated with the commonly used mitochondrial uncoupler like CCCP clearly exposes the many caveats in our understanding of the functioning this organelle. On CCCP treatment, it is seen that the mitochondrial distribution changes from a spread out pattern to a cluster around the nucleus. Why do they cluster around the nucleus? Does this involve ROS mediated signalling between the mitochondria and the nucleus or between the clustered mitochondria themselves? On using a high resolution microscope it is seen that what appears to be a cluster of fused mitochondria is actually made up of many punctuate individual mitochondria. It is known that fission can segregate out damaged or depolarised mitochondria so that it can be removed but what determines the sites of this fission? How does the cell know how much of the mitochondria have to be removed from the electrically continuous mitochondrial network? Are there internal sensors which can sense the local membrane potential within the mitochondria which decide the sites for mitochondrial fission? Once the depolarised mitochondria are separated from the rest by fission, it can undergo mitophagy. How does the cell specifically direct the autophagy machinery to the damaged mitochondria without activating bulk autophagy? And finally, how does the cell decide when to switch from mitochondrial quality control to activation of apoptosis? 

Our knowledge of oxidative stress and mitochondrial biology is still very rudimentary with a lot of unexplored questions. With the advent of sophisticated techniques like super resolution imaging, mass spectrometry based mitochondrial proteome analysis, and new *in vivo* magnetic resonance spectroscopy (MRS) based techniques to analyse mitochondrial function, mitochondrial research is progressing rapidly towards a clearer understanding of the mitochondria and their interaction with the rest of the cell.
